# Screening early numerical development in Brazilian children: adaptation and validation of MARKO-S

**DOI:** 10.3389/fpsyg.2026.1794101

**Published:** 2026-09-17

**Authors:** Fernanda Rocha de Freitas, Moritz Herzog, Mariuche Gomides, Antje Ehlert, Annemarie Fritz, Vitor Geraldi Haase

**Affiliations:** 1 Department of Psychology, Universidade Federal de Minas Gerais, Belo Horizonte, Brazil; 2 Institute for Mathematics Education, University of Cologne, Cologne, Germany; 3 Institute for Inclusive Education, University of Potsdam, Potsdam, Germany; 4 Akademie Wort + Zahl, Cologne, Germany

**Keywords:** assessment, early childhood education, mathematical learning difficulties, numerical development, psychometric validation, screener

## Abstract

Mathematical learning difficulties (MLD) are estimated to affect a substantial proportion of children worldwide, yet psychometrically validated screening instruments for early numerical development remain scarce in non-WEIRD countries, including Brazil. MARKO-Screening is a brief, theoretically grounded screening instrument designed to assess early numerical development in preschool and early elementary children. Based on a developmental trajectory model of numerical concept acquisition, MARKO-S evaluates foundational concepts including counting, ordinality, and cardinality. This study aimed to adapt and validate a Brazilian Portuguese version of MARKO-S (MARKO-S BR) for early identification of children at risk for MLD, examining evidence of content validity, internal structure, and relations with other variables in a sample of 1,200 children from preschool to second grade, enrolled in public and private schools across three Brazilian regions. Results provided evidence of structural validity, with item difficulty largely aligned with the theoretical model, and convergent validity through positive correlations with established mathematical assessments. MARKO-S BR scores showed significant grade-related differences, consistent with the expected acquisition of numerical concepts across schooling. Substantial performance gaps also emerged between public and private school students, reflecting systemic inequalities in early mathematics education in Brazil. These findings support MARKO-S BR as a psychometrically sound, culturally adapted screening instrument, addressing the scarcity of validated early mathematics assessments and providing Brazilian educators and clinicians with an evidence-based digital tool for early identification of children at risk for MLD.

## 1 Introduction

Mathematical knowledge is a fundamental competency for educational success, professional development, and well-being throughout adulthood. This influence extends beyond academic trajectories, encompassing future economic opportunities, health, and life satisfaction (Bjälkebring and Peters, 2021; Heilmann, 2020; Watts, 2020). Early numerical skills have been shown to predict later academic success even after accounting for literacy and cognitive skills (Watts et al., 2014). Despite the critical role of early numerical development, global estimates indicate that more than half of children worldwide fail to reach the minimum level of mathematical proficiency (OECD, 2023; UNESCO, 2025). This challenge is even more acute in low- and middle-income countries, where structural barriers impede equitable access to quality mathematics education. According to the PISA’s assessment, Brazil ranks in the bottom quartile of participating nations, with two-thirds of students performing below the minimum level of mathematical proficiency (OECD, 2023).

Given the impact of early mathematical skills on multiple domains of development, investment in the early identification of children with mathematical learning difficulties (MLD) becomes a priority. The acquisition of arithmetic knowledge follows a hierarchical structure, with foundational concepts such as counting serving as the basis for more advanced skills including arithmetic operations and problem-solving (Clements and Sarama, 2014). When children do not succeed in acquiring fundamental numerical concepts, the disparities in their knowledge tend to amplify over time. As a consequence, subsequent learning becomes increasingly challenging, and existing academic difficulties intensify (Geary et al., 2012). Early identification of children with or at risk for MLD enables the implementation of targeted interventions that can prevent the accumulation of deficits and promote successful development (Gersten et al., 2012).

A growing number of instruments to evaluate early mathematical skills have been developed globally. In a systematic review study, Outhwaite et al. (2024) examined a total of 66 instruments designed for children up to 8 years old, encompassing 41 assessments and 25 screening instruments. These instruments evaluate domains such as number sense, counting, arithmetic operations, and geometric concepts primarily through paper-and-pencil assessments. The instruments’ reliability and validity evidence were evaluated according to established educational measurement standards (American Educational Research Association, American Psychological Association, National Council on Measurement in Education, 2014; Reeves and Marbach-Ad, 2016) to identify those with sufficient psychometric quality for reliably assessing early mathematical skills and differentiating between typically developing children and children with MLD.

Despite the wide array of instruments available, Outhwaite et al. (2024) underscored critical gaps including the insufficient evaluation of established reliability and validity standards. Less than one quarter of the instruments meet acceptable standards across more than two key psychometric domains including structural validity, internal consistency, and criterion validity. Within screening instruments: only one met acceptable thresholds for concurrent validity (Purpura et al., 2015), only three were adequate to identify children at risk for MLD (Chatzaki et al., 2024; Clarke et al., 2011; Erford et al., 1998), only three were adapted and validated cross-culturally in multiple countries and languages (Jordan et al., 2010; Kızıltepe, 2018; Purpura et al., 2015; Tsigilis et al., 2023; Van De Rijt et al., 2003). Notably, more than half of all identified instruments were developed in WEIRD (Western, Educated, Industrialized, Rich, and Democratic) societies, despite the review encompassing tools from over 55 countries and 31 languages (Outhwaite et al., 2024). Consequently, psychometrically validated instruments for the early identification of mathematical learning difficulties remain critically scarce in non-WEIRD countries.

The Brazilian context exemplifies this gap: Outhwaite et al. (2024) identified only three instruments with validated psychometric evidence for assessing early numerical skills, and a complementary search in Portuguese-language databases revealed no additional instruments meeting these criteria. Within these three instruments, the Zareki-R is the only one that has been validated across cultures. It comprises 11 subtasks that evaluate different components of number processing and mental calculation. The instrument demonstrated good internal consistency (Cronbach’s *α* = 0.86) and criterion validity, as evidenced by weak to moderate correlations (0.41–0.64) to other mathematical measures (Santos et al., 2012). The School Achievement Test – Second Edition (SAT-II) was designed for curriculum-based school achievement in numbers and operations and showed excellent internal consistency (Cronbach’s α = 0.97), however, other types of validity evidence were not reported (Viapiana et al., 2016). The Number Transcoding Task is the only available screening instrument, and it focuses exclusively on number writing. Moura et al. (2015) reported excellent internal consistency (Kuder–Richardson–20 = 0.91) and adequate diagnostic accuracy for first- and second graders (AUC = 0.79 and 0.97 respectively). Importantly, none of the three instruments provided evidence of structural validity. The absence of validated screening instruments for the comprehensive evaluation of numerical skills remains a critical obstacle to the early identification of children at-risk for MLD in Brazil.

Among instruments designed to assess early numerical conceptual development, the MARKO-Diagnostic (MARKO-D) stands out as a comprehensive paper-and-pencil assessment for children aged 4 to 8 years (Ricken et al., 2013). MARKO-D is grounded in a trajectory model of number concept development (Fritz et al., 2013) which describes numerical knowledge acquisition as overlapping waves of increasingly sophisticated understanding of numbers and their relations. The model proposes six hierarchical levels of development, each characterized by the acquisition of a key numerical concept that builds upon earlier conceptual understanding. The mastery of each developmental level is considered when a child successfully completes at least 75% of the tasks at that level. This theoretically grounded approach enables not only summative assessment of risk status for MLD, but also formative identification of the child’s current developmental level and specific conceptual gaps that require further instruction.

According to Fritz et al. (2013), these levels reflect children’s progressive understanding of: the number sequence and enumeration of small sets through one-to-one correspondence (Level I - Counting); numbers as ordered positions on a mental number line, enabling identification of predecessors and successors, comparison of magnitudes based on positional order, and performance of simple arithmetic through counting strategies (Level II - Ordinal Number Line); numbers as representations of specific quantities and as composite units decomposable into smaller parts, allowing addition and subtraction to be understood as combining or separating sets (Level III - Cardinality); the relationship between a number as a whole and its constituent parts, allowing flexible set decomposition and resolution of problems involving a missing part (Level IV - Part-part-whole relations); numbers as representations of distance between other numbers, allowing comparison of how much larger or smaller one number is in relation to another (Level V - Equidistant number line intervals); and the concept that numbers can be divided into equal parts, forming the foundation for multiplication, division, and place value concepts (Level VI - Units in numbers; see Figure 1).

**Figure 1:**
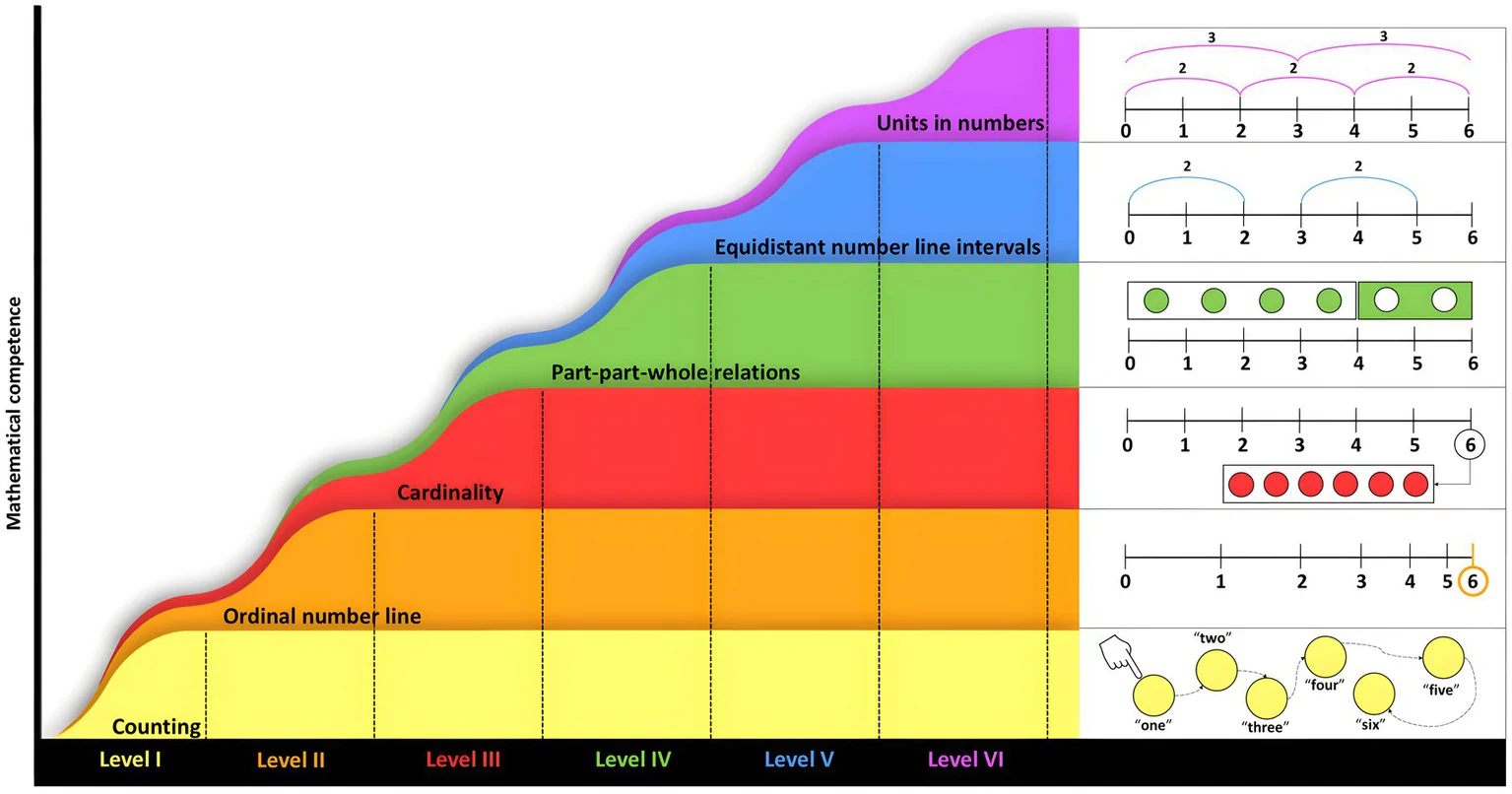
Trajectory model of number concept development (adapted from Fritz et al., 2013). The colored waves (left) represent the hierarchical sequence of levels, from Counting to Units in Numbers. The panels (right) illustrate the child’s corresponding representation of the number 6 at each level: (I) Enumeration of a set of 6 objects through one-to-one correspondence; (II) The number 6 placed at the end of the line indicates it is larger, but the line is still logarithmically compressed; (III) With cardinality, 6 now represents the whole set of 6 objects; (IV) 6 can be decomposed into parts (e.g., 2 and 4); (V) The number line has equidistant intervals, so the distance between 0 and 2 equals the distance between 3 and 5; (VI) 6 can be grouped in different ways into equal sets (e.g., 3 groups of 2, or 2 groups of 3).

The hierarchical structure of the numerical conceptual levels proposed by the conceptual model has been empirically validated through the MARKO-D instrument, which assesses the first five levels of the model (Fritz et al., 2013, 2018). The original German assessment demonstrated robust psychometric properties including strong structural validity, excellent internal consistency (Cronbach’s *α* = 0.91), test–retest reliability (0.89), and strong concurrent validity (*r* = 0.77; Ricken et al., 2013). The MARKO-D instrument has been successfully adapted and validated across diverse cultural contexts, including four South African languages (English, Afrikaans, isiZulu, and Sesotho; Henning et al., 2021), and most recently, Spanish within the Chilean context (Espinoza et al., 2025). These adaptations establish substantial measurement invariance across different populations and languages, providing robust structural validity of the conceptual model across diverse contexts, including non-WEIRD countries.

Complementing the comprehensive MARKO-D assessment, the MARKO-Screening (MARKO-S) was developed as a brief screening instrument also grounded in the trajectory model of number concept development focused on the three first levels. Early identification of children who have not adequately acquired the foundational numerical concepts evaluated by MARKO-S is essential, as mastery of counting procedures, ordinal relationships, and cardinality forms the basis upon which all subsequent arithmetic reasoning and efficient calculation strategies are built (Geary et al., 2019; Gelman and Gallistel, 1978). Children who struggle to progress through these developmental levels face the risk of persisting in inefficient counting-based strategies, failing to understand part-whole relationships, and experiencing cumulative learning deficits (Krenger and Thevenot, 2025; Mazzocco et al., 2013). MARKO-S can be administered in approximately 15 min via an online platform, facilitating rapid identification of children at risk for MLD in preschool and early primary school settings.

The original version of MARKO-S demonstrated strong diagnostic accuracy for identifying children at risk for MLD, with sensitivity of 0.86 and specificity of 0.76 six months before school entry, and sensitivity of 0.79 and specificity of 0.96 in the beginning of first grade (Ehlert et al., 2020). Additionally, the instrument exhibits adequate person reliability (0.69), and strong correlation with the MARKO-D (*r* = 0.778), with unidimensional Rasch model confirming that the three theoretical competence levels are empirically distinct and ordered hierarchically (Ehlert et al., 2020). Gürsoy et al. (2020) validated a Turkish version of the MARKO-S within a German-Turkish context, where bilingual children were assessed using both versions of the instrument. Comparative analyses showed that items operationalizing the same arithmetic concepts in Turkish and German presented similar item difficulty values, validating the cross-linguistic stability of the conceptual model also for the screening instrument. Given this theoretical grounding, its brief digital administration format, and the cross-cultural stability of both the model and the instrument demonstrated in previous studies, MARKO-S is considered a suitable option for early identification of children at risk for MLD in the Brazilian context.

This study aims to adapt and validate a Brazilian Portuguese version of the MARKO-S (MARKO-S BR) for early identification of children at risk for MLD, testing validity evidence of the screening instrument. Specifically, three main sources of validity evidence will be investigated: (1) Evidence based on content: systematic translation, cultural adaptation, and expert evaluation to ensure semantic and conceptual equivalence with the original German version; (2) Evidence based on internal structure: testing whether the hierarchical organization of the three conceptual levels of the Trajectory Model remains supported in the Brazilian population, and verifying internal consistency through Rasch and Classical approaches; and (3) Evidence based on associations with other variables: establishing correlations with external measures of numerical cognition, mathematical abilities, and academic achievement; examining whether the instrument captures expected performance differences across developmental stages and educational contexts. By establishing comprehensive validity evidence, this study provides an evidence-based screening tool for early identification of numerical conceptual development in Brazilian educational and clinical contexts.

## 2 Methods

### 2.1 Participants

The sample consisted of 1,200 children with typical cognitive abilities (scoring above the 5th percentile on the Raven’s Colored Progressive Matrices; Paula et al., 2018). Participants were enrolled in the final year of preschool (*n* = 352, *M_age_* = 70.76 months, SD = 3.87, 55.10% girls), first grade (*n* = 445, *M_age_* = 77.91 months, SD = 4.12, 53.3% girls), and second grade (*n* = 403, *M_age_* = 89.84 months, SD = 4.18, 50.6% girls). Participants were drawn from both public (48.20%) and private (51.80%) schools. Although public schools serve approximately 80% of Brazilian students in basic education, the sample was intentionally balanced between public and private schools to ensure adequate statistical power for between-group comparisons. Schools were located in three different Brazilian cities: Belo Horizonte (Southeast region; 34.80%), Porto Alegre (South region; 32.70%), and Vitória da Conquista (Northeast region; 32.60%). The distribution of participants by grade and school type within each city is detailed in Table 1. While the original MARKO-S in Germany targets preschoolers and first graders, the adaptation was expanded to include second graders based on pilot studies’ findings described in the Procedures section.

**Table 1:** Participants’ demographic information.

Grade	*N*	Age	Girls (%)	Public school (%)
Belo Horizonte (Southeast region)				
Preschool	107	71.42 (3.77)	54.20%	40.20%
First grade	149	78.08 (3.79)	55.70%	43.60%
Second grade	161	90.19 (3.31)	44.70%	45.30%
Porto Alegre (South region)				
Preschool	126	70.71 (4.05)	53.20%	54.00%
First grade	148	78.25 (3.74)	50.70%	54.10%
Second grade	117	90.81 (4.05)	53.80%	49.60%
Vitória da Conquista (Northeast region)				
Preschool	119	70.21 (3.71)	58.00%	52.10%
First grade	148	77.39 (4.73)	53.40%	50.00%
Second grade	125	88.47 (4.92)	55.20%	44.00%

*N* = total number of participants in each subgroup. Age is reported in months, with mean and standard deviation (SD) presented in parentheses. Percentages of girls and public-school enrollment were calculated within each subgroup (i.e., by grade, within each city).

### 2.2 Materials

Standardized instruments assessing general cognitive abilities and various components of numerical competence were administered. These instruments measured both domain-general and domain-specific abilities relevant to numerical development, providing evidence for the convergent validity of the MARKO-S.

*MARKO-Screening BR*: Grounded in the trajectory model of number concept development (Fritz et al., 2013) it evaluates the first three levels: (I) Counting, (II) Ordinal number line, and (III) Cardinality. The instrument consists of 24 tasks in total, of which 21 are scored items and 3 are practice items. The scored items are evenly distributed across the three levels, with 7 items per level. Each level is considered mastered when the child correctly answers at least 5 of the 7 items, indicating sufficient conceptual understanding of that stage. The assessment is administered digitally through Hogrefe’s HTS-5 online testing platform and takes approximately 15 min. In this study, the MARKO-S was delivered via tablet devices (10.5-inch screen, Android 13 operating system). To minimize potential examiner effects and ensure standardized test administration across all participants, task instructions were presented via pre-recorded audio narration by a voice of neutral acoustic characteristics. The instrument was originally developed in German (Ehlert et al., 2020) and in the current study the adaptation and validation for Brazilian Portuguese is presented.

*Raven’s Colored Progressive Matrices (RCPM)*: A widely used measure of nonverbal reasoning and general cognitive ability. The test consists of 36 pictorial pattern-completion items organized into three sets of increasing difficulty, requiring perceptual analogical reasoning (Paula et al., 2018). Each item shows an incomplete figure, and children choose from six options the one that best completes it. It is an untimed assessment but typically requires 15 to 20 min. The RCPM demonstrated good internal consistency in the present sample, with a Cronbach’s alpha of 0.84.

*Numeracy Screener*: A brief screening instrument assessing symbolic and nonsymbolic numerical magnitude comparison skills (Nosworthy et al., 2013). The task comprises two timed blocks of 1 min each, with 56 items per block. In the symbolic block, children choose which of the two Arabic digits is larger and in the non-symbolic block, children choose which of the two dot arrays has more dots. Each block was preceded by examples and practice items. Numeracy Screener demonstrated excellent internal consistency in the present sample, with a Cronbach’s alpha of 0.95 for the symbolic block and 0.94 for the non-symbolic block.

*Test of Early Mathematics Ability – Third Edition (TEMA-3)*: designed to evaluate mathematical knowledge in children aged 3 years to 8 years (Ginsburg and Baroody, 2003). The test includes 72 items that progress from easy to difficult, organized in two broad domains: informal mathematics (e.g., number comparisons and calculation with concrete objects) and formal mathematics (e.g., Arabic number transcoding and carrying operation). Administration follows baseline and ceiling rules to optimize testing time that typically requires from 35 to 45 min. The TEMA-3 demonstrated excellent internal consistency in the present sample, with a Cronbach’s alpha of 0.97.

*School Achievement Test – Second Edition (SAT-II)*: a curriculum-oriented standardized assessment designed to evaluate fundamental academic skills in Brazilian children from 1st through 9th grade. SAT-II is composed of three subtests: reading, writing and arithmetic, and only the latter was assessed in the current study with first and second graders (Viapiana et al., 2016). This subtest has two grade-specific versions, and the version for first to fifth graders was used in the present study. It evaluates mathematical achievement through 37 items organized in ascending order of difficulty, assessing skills such as counting, number comparison, number line positioning, Arabic numeral writing and basic operations. The test is untimed but typically requires 20 to 30 min. The SAT-II arithmetic subtest showed good internal consistency in the present sample with a Cronbach’s alpha of 0.86.

*Basic Arithmetic Operations Task (BAOT)*: assesses children’s fluency in solving arithmetic calculations involving addition, subtraction and multiplication operations (Gomides et al., 2021a). For this study, only addition and subtraction were assessed in first and second graders. For each operation, two blocks were administered: simple operations with 12 items involving numbers up to 10 (e.g., 5 + 2, 9–5) and complex operations with 15 items involving numbers up to 17 (e.g., 9 + 3, 15–6). Each block has a time limit of 1 min. The BAOT demonstrated good internal consistency in the present sample, with a Cronbach’s alpha of 0.91 for the simple addition block, 0.84 for the complex addition block, 0.90 for the simple subtraction block and 0.78 for the complex subtraction block.

### 2.3 Procedures

The adaptation of MARKO-S for the Brazilian population was conducted following the guidelines of the International Test Commission (International Test Commission, 2017) to ensure content validity and semantic-conceptual equivalence. The initial adaptation phase included translation from the original version in German to Brazilian Portuguese, back-translation to German by two independent bilingual experts to verify correspondence with the original version and expert panel review to address any discrepancies. MARKO-S BR was subsequently tested with a small group of children from different regions of Brazil to ensure task comprehension and make minor adjustments to improve clarity and understanding.

After the initial adaptation phase, MARKO-S BR was assessed in three empirical pilot studies with a total of 414 children from public schools of Belo Horizonte (Mage = 81.11 months; SD = 11.85, 44.44% girls) distributed across the final year of preschool, first grade, and second grade. These pilot studies were designed to verify whether the adapted items were clearly understood and culturally appropriate for the target population, to provide preliminary evidence of item functioning, and to determine the appropriate grade range for the normative sample.

Based on prior evidence that Brazilian children struggle to achieve fluency in basic arithmetic even by the end of elementary school (Gomides et al., 2021b), it was hypothesized that second graders would still show limited mastery of the more advanced concepts assessed by MARKO-S, particularly cardinality (Level III). Consistent with this hypothesis, second graders in all three pilots showed low accuracy on Level III items (ranging from 31.12 to 47.55%), despite outperforming first graders and preschoolers. Based on this pattern, it was decided to extend the normative sample to include second-grade children, given that the instrument retained sufficient sensitivity to capture developmental variability beyond the age range originally targeted by the German version.

After each pilot study, Rasch analysis’ results and descriptive accuracy data were examined to refine item content and ensure that the level hierarchy proposed by the trajectory model of number concept development was empirically preserved. Exclusions and modifications of items in the Brazilian adaptation were data-driven, focusing on resolving misalignments with the expected developmental level structure and ensuring psychometric adequacy within the Rasch framework. New or revised items in the Brazilian version were variations of existing original items or represent changes in scoring, such as the inclusion of a previously non-scored item. Additionally, a new example was added to the “Judge equality of sets with distinct spatial arrangements” item to address potential priming effects and enhance clarity. The final version consisted of 21 items (plus 3 example items), with seven items per developmental level, ensuring at least five anchor items per level to maintain measurement equivalence with the original instrument. Descriptions of the item-level modifications for each developmental level are provided in Table 2.

**Table 2:** Overview of item-level modifications for the MARKO-S BR by developmental level.

Item	Item description	MARKO-S BR
Level I - Counting		
7	Subitize 2 (t)	Original item
8	Subitize 3 (t)	Original item
13	Give 3 dots	Original item
14	Give 7 dots	Original item
15	Create a set of equal size: 5 dots	Original item
4	Count how many: 5 dots	Original item
18	Show 5 fingers	Incorporated in the score
-	Detect an error in the number sequence (1, 2, 3, 5)	Excluded due to difficulty misalignment (hard)
Level II - Ordinal number line		
1	Identify the number before 3	Original item
2	Identify the number between 3 and 5	Original item
16	Indicate the flower in the 6th position	Original item
17	Indicate the flower in the 4th position	New variation
3	Choose the smaller number: 8 or 7	Original item
11	Solve a simple addition word problem: 3 + 2	Original item
12	Solve a simple subtraction word problem: 7–2	New variation
-	Choose the number closest to 4: 2 or 5	Excluded due to poor Rasch model fit (infit > 1.33)
-	Choose the larger number: 6 or 7	Excluded due to difficulty misalignment (easy)
Level III - Cardinality		
6	Judge equality of sets with distinct spatial arrangements (t) (e)	Original item (included one more example)
9	Groupitize 7 (t)	Original item
10	Groupitize 8 (t)	New variation
5	Determine the missing quantity in an increasing sequence (e)	Original item
19	Decompose 5 with fingers	Original item
20	Create a set with 2 fewer elements than 7	Original item
21	Create a set with 3 more elements than 5	Original item

(t) The stimuli appeared with a time limit of 2 s, (e) item preceded by an example item.

In the final validation phase, MARKO-S BR and other standardized instruments were administered individually to a large sample of Brazilian children from June to November (middle to the end of Brazilian school year). Written informed consent was obtained from parents or legal guardians, and oral assent from children was secured prior to testing. The study received approval from the local Research Ethics Committee. This data collection aimed to gather comprehensive evidence of construct validity focusing on the internal structure of the test and its relationships to relevant external variables.

### 2.4 Statistical analysis

A one-parameter logistic (1PL) Rasch model for dichotomous data was conducted to examine the psychometric properties of MARKO-S BR using Winsteps® (Version 5.10.4: Linacre, 2025; Rasch, 1960). All 21 items of the instrument (described in Table 2) were entered into the model. Considering that no missing data were present in the final dataset, no imputation or case-exclusion procedures were required. The analysis followed a sequential four-step procedure. First, unidimensionality was examined through two complementary approaches. At the Rasch level, Principal Components Analysis (PCA) of standardized residuals was used to verify whether the first contrast remained below the conventional threshold of 5% of unexplained variance (Smith, 2002). In parallel, a bifactor model was estimated in R (Version 4.6.0; R Core Team, 2026), with one general factor and three specific factors corresponding to the conceptual levels of MARKO-S BR was estimated, and bifactor indices were inspected, including explained common variance (ECV) and omega hierarchical (*ω*H; Reise et al., 2018; Rodriguez et al., 2016). Second, item fit was evaluated through infit and outfit mean square (MNSQ) statistics within the conventional acceptable range from 0.7 to 1.3 (Linacre, 2002). Infit statistics evaluate the appropriateness of responses from individuals whose ability level closely matches the item’s difficulty, whereas outfit statistics assess response irregularities from individuals whose ability substantially deviates from the item’s difficulty level. Third, the developmental hierarchy across the three conceptual levels evaluated by MARKO-S BR was examined by comparing item difficulties calibrated within the Rasch model (Bond et al., 2020). Fourth, person and item reliability and separation indices were calculated to determine measurement precision in differentiating performance levels, with person reliability values ≥ 0.70 considered adequate (Bond et al., 2020).

In addition to the Rasch-based reliability estimates, internal consistency was evaluated using classical test theory approaches, conducted in R. McDonald’s Omega (ω) was used as the primary estimate, with Cronbach’s Alpha (*α*) also reported for comparability with previous research. Acceptability was determined by values exceeding 0.80, following recommendations for educational screening instruments (American Educational Research Association, American Psychological Association, National Council on Measurement in Education, 2014).

Convergent validity was examined through Pearson correlations between MARKO-S BR and independent measures assessing general cognitive ability and numerical cognition, including nonverbal reasoning (Raven’s Colored Progressive Matrices), symbolic and non-symbolic numerical magnitude comparison (Numeracy Screener), broad mathematical ability (TEMA-3), curriculum-based arithmetic achievement (SAT-II arithmetic subtest), and basic arithmetic fluency (BAOT simple and complex addition and subtraction subtests). To control for the expected effect of grade-level progression on raw performance, all variables entered into the correlational analyses were first converted into z-scores calculated separately within each grade. All correlational analyses were conducted in R, as described above. Pairwise deletion was applied for missing data, so that each correlation was calculated using only the cases with valid scores on both variables involved.

Known-groups validity was examined through a one-way between-subjects analysis of variance (ANOVA), conducted in R, comparing MARKO-S BR total raw scores across the three grade levels (preschool, first grade, second grade). Effect size for the ANOVA was estimated as eta-squared (η^2^) with a 95% confidence interval. Given that the ANOVA was statistically significant, pairwise *post-hoc* comparisons between the three grades were conducted using Bonferroni-corrected pairwise *t*-tests. To evaluate school-type effects independent of grade-level differences, MARKO-S BR total scores were standardized into grade-based z-scores, as described above. Independent-samples t-tests, with Welch’s correction for unequal variances, then compared these standardized scores between children enrolled in public versus private schools. Effect size for each t-test was estimated as Cohen’s d with a 95% confidence interval.

## 3 Results

### 3.1 Structure validity

The developmental model of number concepts underlying MARKO-S and previous studies with the screening instrument postulates that children’s numerical concepts development can be represented as a single latent dimension (Ehlert et al., 2020). To verify this assumption and the applicability of Rasch model for the current data, the dimensionality of the data was assessed using Principal Components Analysis (PCA) of the standardized residuals and complemented by a bifactor analysis to examine the overall strength of the general dimension. The PCA revealed that the Rasch model accounted for 47.9% of the total raw variance in responses, and the first contrast had an eigenvalue of 1.69, accounting for only 4.2% of the unexplained variance, thereby supporting the unidimensionality of MARKO-S. The bifactor analysis further strengthened the evidence for an essentially unidimensional structure of MARKO-S, with the general factor accounting for 65.3% of the common variance (ECV = 0.653) and showing a high omega hierarchical value (ωH = 0.78). In contrast, the level-specific factors showed low omega hierarchical values (Level I: ωH = 0.40; Level II: ωH = 0.07; Level III: ωH = 0.18), indicating limited reliable variance beyond the general dimension.

Another important assumption underlying MARKO-S is that items assessing more foundational numerical concepts should be easier than those assessing more advanced ones, reflecting the hierarchical structure of the trajectory model and its invariance across different countries. Consistent with the conceptual model and previous studies, items representing Level I were expected to be easier than those assessing Level II which were expected to be easier than those items assessing Level III. To examine whether this hierarchical ordering of item difficulties was preserved in the Brazilian sample, a Rasch analysis was conducted. The hierarchical ordering of item difficulties confirmed the expected difficulty ordering across conceptual levels except for item 16 (Indicate the flower in the 6th position) from Level II that was more difficult than item 5 from Level III (Determine the missing quantity in an increasing sequence). Detailed item difficulty parameters are presented in Table 3.

**Table 3:** Rasch item parameters and model-fit indices for the MARKO-S BR items.

Item	Level	Difficulty	Standard error	Infit	Outfit
21	III	2.77	0.08	0.83	0.80
20	III	2.67	0.08	0.83	1.05
06	III	2.17	0.07	1.22	2.92
19	III	2.09	0.07	1.11	1.18
10	III	1.95	0.07	1.16	1.26
09	III	1.73	0.07	1.21	1.35
16*	II	1.23	0.07	0.89	0.89
05*	III	1.00	0.08	1.05	1.05
12	II	0.77	0.08	0.87	0.81
02	II	0.26	0.08	0.82	0.68
17	II	0.20	0.08	0.74	0.64
11	II	−0.05	0.08	0.78	0.59
01	II	−0.26	0.09	0.93	0.95
03	II	−1.02	0.10	0.95	0.79
15	I	−1.11	0.10	1.00	1.05
14	I	−1.42	0.11	0.97	1.61
18	I	−1.47	0.11	1.18	2.24
04	I	−2.43	0.15	1.06	2.58
13	I	−2.54	0.15	0.94	1.39
08	I	−3.13	0.19	0.88	2.46
07	I	−3.37	0.21	0.98	1.71

Level = hierarchical level according to the Fritz et al. (2013) model (I = Counting, II = Ordinal Number Line, III = Cardinality). Infit and Outfit are reported as mean-square (MNSQ) statistics, with values close to 1.0 indicating good fit to the Rasch model. *Items 16 (Level II) and 5 (Level III) deviated from the hierarchical difficulty order expected by the theoretical model.

The Rasch item fit statistics demonstrated a satisfactory model fit. Infit mean square (MNSQ) values were within the expected range of 0.7 to 1.3 for all 21 items, indicating consistent predictability of responses across the instrument. Regarding outfit mean square (MNSQ), 10 of the 21 items were within the acceptable interval, while eight items were above 1.30 and three were below 0.70. A closer inspection revealed that six of the eight items with elevated outfit statistics were significantly easier relative to the sample. This is a common pattern consistent with Karabatsos’ (2000) observation that outfit statistics are sensitive to outliers, particularly when high-ability examinees respond unexpectedly to easy items. Among the items with elevated outfit values, Item 6, “Judge equality of sets with distinct spatial arrangements” from Level III, showed the highest value and may reflect a different mechanism: as a dichotomous item, it affords a 50% probability of a correct response by chance, making it susceptible to unexpected correct answers from lower-ability examinees (Levels I and II), which the outlier pattern outfit is designed to detect. Despite this, infit values for all eleven items remained within acceptable limits, indicating that none of these items substantially distorted the overall measurement structure, and supporting their retention in the model. The person-item map illustrates that the distribution of item difficulty levels adequately covers the range of participant abilities. The item-person map shows that, overall, the sample exhibited a higher level of the latent trait compared to the average item difficulty (mean person ability = 1.81; mean item difficulty = 0; Figure 2). Considering that MARKO-S is a screening instrument designed to identify children at risk for MLD, this result is compatible with the instrument’s aim of being more sensitive.

**Figure 2:**
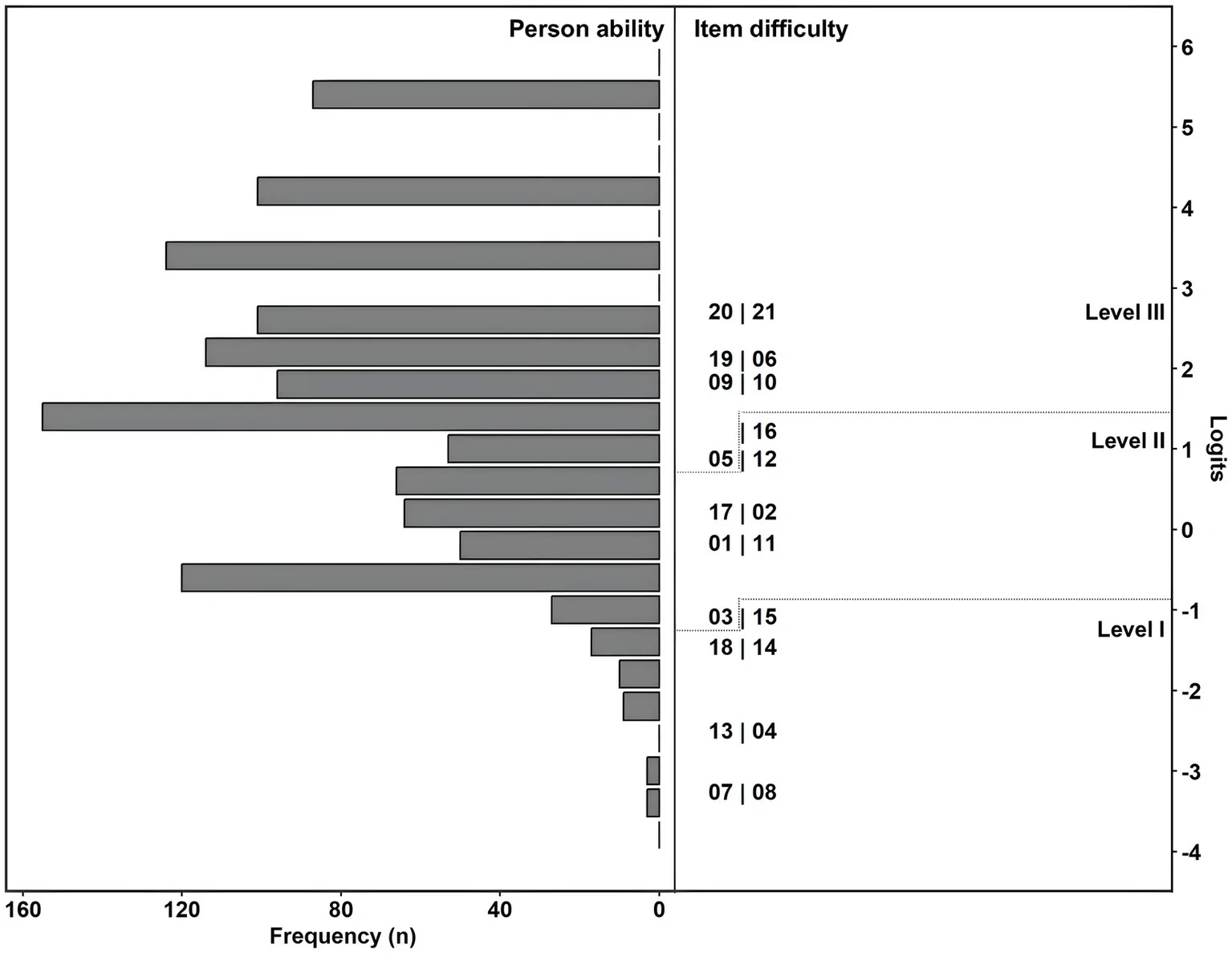
Wright map of person ability distribution and item difficulty for MARKO-S BR. The left panel shows the absolute frequency (n) of participants’ ability along the logit scale. The panel on the right shows the location of each item (numbered) according to its difficulty, also expressed in logits. Both panels share the same vertical scale, allowing direct comparison between person ability and item difficulty. Dashed horizontal lines indicate the thresholds separating Level I - Counting (−3.37 to −1.11 logits), Level II - Ordinal number line (−1.02 to 1.23 logits), and Level III - Cardinality (1.00 to 2.77 logits).

### 3.2 Internal consistency

Internal consistency and reliability of the MARKO-S BR were evaluated through complementary analytical approaches. Within the Rasch measurement framework, person reliability was 0.79 and the separation index was 1.94, indicating that the instrument can reliably differentiate at least two levels of individual performance. Item reliability was 1.00, with a separation index of 17.15, confirming that the sample size was sufficient to precisely estimate item difficulties and support the hierarchical ordering of items. Based on classical test theory, the instrument demonstrated excellent internal consistency, evidenced by McDonald’s *ω* of 0.944 and Cronbach’s *α* of 0.940, supporting the reliability of the items in measuring the intended construct of early numerical conceptual development.

### 3.3 Convergent validity

Pearson correlations were computed between the MARKO-S and a range of external measures representing mathematical, general cognitive ability, and academic achievement, with scores on all measures standardized as z-scores within each school grade to account for grade-related variance (see Table 4). The MARKO-S BR exhibited a moderate correlation with nonverbal reasoning measured by the Raven’s Colored Progressive Matrices, suggesting an important association between general cognitive ability and early mathematical concept acquisition. Regarding numerical magnitude comparison, MARKO-S was weakly correlated with the non-symbolic Numeracy Screener and was moderately correlated with the symbolic block. A strong correlation was observed between the MARKO-S and the TEMA-3, underscoring the shared variance between the two comprehensive early mathematics instruments. MARKO-S also demonstrated a moderate correlation with the arithmetic subtest of the SAT-II and with basic arithmetic fluency tasks from the BAOT.

**Table 4:** Pearson correlation between MARKO-S BR and external measures.

External measures	*n*	MARKO-S BR
Raven’s colored progressive matrices	1,200	0.537*
Numeracy Screener – Non-symbolic	1,157	0.290*
Numeracy Screener – Symbolic	1,159	0.476*
TEMA-3	891	0.725*
School Achievement Test - II	848	0.699*
BAOT - Simple Addition	844	0.609*
BAOT - Simple Subtraction	844	0.483*
BAOT - Complex Addition	844	0.496*
BAOT - Complex Subtraction	844	0.402*

TEMA-3, Test of early mathematics ability, 3rd edition; BAOT, Basic arithmetic operations test. *All correlations were significant at p < 0.001.

### 3.4 Known-groups comparison

A one-way ANOVA revealed a significant effect of school grade on MARKO-S total scores, *F*(2, 1,197) = 276.15, *p* < 0.001, η^2^ = 0.32, 95% CI [0.28, 1.00], indicating a large effect size. *Post hoc* comparisons confirmed significant grade-related differences: second graders (M = 17.77, SD = 2.94) scored higher than first graders (M = 14.94, SD = 3.86), who outperformed preschoolers (M = 11.49, SD = 4.12). Analysis of children’s distribution across the theoretical levels of number concept development evaluated by MARKO-S BR further illustrates these grade-related differences (Figure 3). In the final year of preschool, the majority of children were concentrated at Level II (55.1%), in first grade, the proportion of children at Level III increased substantially (45.8%) and in the second grade, most children had reached Level IV or higher (55.8%).

**Figure 3:**
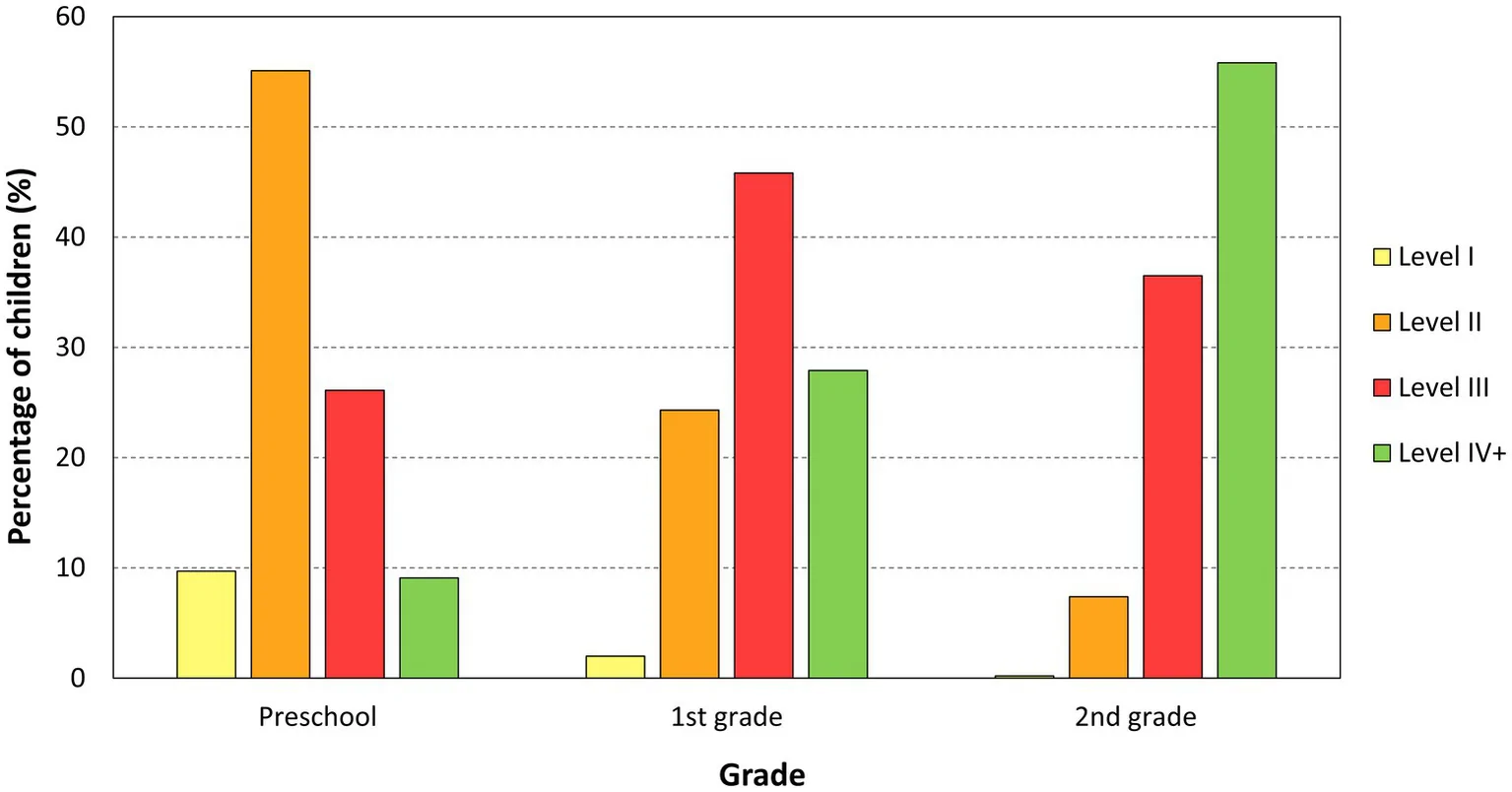
Distribution of children by grade across the levels evaluated by MARKO-S BR. Percentages of children per level were calculated considering the total number of children within each grade as 100%. Level I = Counting; Level II = Ordinal Number Line; Level III = Cardinality. Level IV + represents children who have mastered the three first conceptual levels, limiting the instrument’s capacity to differentiate their current developmental level.

A comparison of performance between children of public and private schools revealed significant differences in early mathematical skills. Children attending private schools outperformed those in public schools on the MARKO-S, controlling for grade-level effects, t(1128) = 13.03, *p* < 0.001, d = 0.75, 95% CI [0.64, 0.87], indicating a large effect size.

## 4 Discussion

The present study aimed to adapt and validate the Brazilian Portuguese version of MARKO-S, a screening instrument designed for early identification of children at risk for MLD. Evidence of validity was evaluated based on test content, internal structure, and relations with other variables. The adaptation process followed the International Test Commission guidelines (International Test Commission, 2017) ensuring that the MARKO-S BR items measure the same numerical concepts as the original German screening instrument (Ehlert et al., 2020). The results provide strong evidence supporting the validity and reliability of MARKO-S BR for screening early numerical skills in Brazilian children from preschool to second grade.

According to the Rasch Model analysis, the items showed overall low difficulty, with most children demonstrating ability levels above the average item difficulty. The distribution of items across the ability range reflects parameters that are ideal for screening instruments, which prioritize sensitivity over specificity: Typically developing children tend to score above average while the instrument remains sensitive to lower-performing children at-risk for MLD (Jenkins et al., 2007; Klingbeil et al., 2019). This pattern was particularly evident among second-grade children, who achieved notably high accuracy across most items, consistent with the instrument’s original design for preschool and early first-grade populations. It is worth noting that the pilot studies informing the decision to extend the normative sample to second grade included only public-school children, who showed limited mastery of Level III concepts. In the final normative sample, however, second-grade children from private schools performed considerably better, approaching a ceiling effect on the instrument. This suggests that while MARKO-S BR retains adequate sensitivity for identifying at-risk second graders, particularly in public school settings, its utility for differentiating among higher-performing second graders, especially in private schools, may be more limited.

The items’ infit and outfit were adequate for the screening instrument, indicating that their difficulty parameters are estimated with precision and that items contribute meaningfully to the Rasch model. Nonetheless, Item 6 showed the highest outfit value attributable to its dichotomous format, which affords a 50% probability of a correct response by chance among lower-ability examinees. Future studies using MARKO-S or similar instruments should give particular attention to dichotomous items with chance-level guessing probability, as these may show outfit inflation unrelated to construct misfit. Furthermore, based on classical test theory, the instrument demonstrated high internal consistency, providing additional evidence of the reliability of the MARKO-S BR. Taken together, these findings indicate that the MARKO-S BR is a reliable tool for assessing early numerical abilities.

The hierarchical organization of item difficulty proposed by the conceptual model can be found in the MARKO-S BR adaptation. Across the three initial levels of numerical development, items assessing more basic levels were consistently easier than those assessing more advanced levels. One exception was observed in Item 16 (Level II), which required identifying the 6th position in a row of flowers that was more difficult than Item 5 (Level III). This deviation appears to be a linguistic artifact rather than a curriculum-related effect. The ordinal word “sexto” (6th) bears little phonological resemblance to its cardinal counterpart “seis” (six) and has unrelated homophonous meanings in Portuguese, unlike German “sechste”/"sechs.” Consistent with this explanation, Item 17, which required identifying the 4th position using “quarto” (closely resembling the cardinal “quatro”), showed comparatively low difficulty. This contrast between two items of the same task type suggests the violation is specific to the lexical form “sexto” rather than a broader failure of the hierarchical model, which was otherwise preserved across all other item comparisons. Future adaptations should consider alternative position-naming items with clearer cardinal-ordinal correspondence in Portuguese.

Overall, the hierarchical ordering of item difficulty across developmental levels was consistent with previous validations of MARKO instruments in Germany (Ehlert et al., 2020), South Africa (Henning et al., 2021), Chile (Espinoza et al., 2025), and bilingual German-Turkish (Gürsoy et al., 2020), demonstrating the model’s cross-cultural applicability. The common conceptual model underlying MARKO instruments enables consistent measurement and comparable findings across populations, facilitating the understanding of early mathematical development.

MARKO-S BR demonstrated appropriate convergent validity through positive correlations with external measures of mathematical and cognitive performance. The moderate correlation with nonverbal reasoning confirmed shared variance with general cognitive ability, while also indicating that MARKO-S BR captures domain-specific mathematical competencies beyond general intelligence. Correlations with established early mathematics measures (TEMA-3 and Numeracy Screener) provided evidence that MARKO-S BR effectively captures foundational mathematical skills. Regarding numerical magnitude processing, a weak association with non-symbolic numerical representations contrasted with a moderate relation to a symbolic numerical representation, consistent with findings indicating that symbolic number representation proficiency is more predictive of arithmetic knowledge than non-symbolic magnitude understanding (Li et al., 2018; Lyons et al., 2014). Moreover, moderate correlations with measures assessing more complex arithmetic operations (BAOT and SAT-II) demonstrated that MARKO-S BR relates to both arithmetic fluency and mathematical school achievement, consistent with developmental models (Krajewski and Schneider, 2009).

Children’s performance on MARKO-S BR showed significant grade-related differences, with higher scores consistently associated with more advanced school grades. Given the cross-sectional design of this study, however, these differences provide only suggestive evidence that older children have acquired more advanced numerical concepts, and cannot establish that individual children follow this trajectory over time. Longitudinal data would be essential to determine whether the grade-related patterns observed reflect individual developmental change in the Brazilian sample. A grade-related pattern was observed in the distribution of children across MARKO-S BR conceptual levels. Specifically, a greater concentration of children was observed at the ordinal number line level in preschool, with increasing proportions at cardinality and higher levels in first and second grade. However, as MARKO-S BR targets identification of children who have not acquired cardinality by school entry and are at risk for MLD, this distribution of children across levels warrants attention. Approximately one-quarter of first graders were not yet acquiring cardinality and nearly half of second graders had not yet mastered the three foundational levels. Failure to consolidate foundational concepts at school entry aligns with a cascading trajectory of mathematical gaps documented in Brazilian education: Children in upper elementary grades continue struggling with basic arithmetic fluency (Gomides et al., 2021b), and more than half of Brazilian students reach the end of middle school without achieving the minimum level of mathematical proficiency necessary for participation in everyday life (OECD, 2023; UNESCO, 2025).

The substantial proportion of children failing to acquire essential mathematical knowledge becomes particularly acute when examining inequalities between public and private schools in Brazil. In the present study, children attending public schools demonstrated lower performance on MARKO-S BR than their private school peers, with a large effect size. This performance gap reflects systemic inequalities embedded in Brazil’s dual educational system. Private schools serving higher-SES families provide more structured educational support, while public schools serving lower-income populations are commonly constrained by inadequate teacher preparation, limited resources, and working conditions that are obstacles to effective instruction (Menezes et al., 2016). Given these substantial differences, separate norms by school type need to be established for MARKO-S BR, consistent with the common practice among Brazilian educational instruments, to ensure developmentally appropriate expectations within each educational context. Critically, our results show that this gap emerges at or before school entry, reinforcing the critical importance of early numerical practices. Children who have access to structured early mathematics instruction and numeracy-enriched home environments are more likely to consolidate foundational concepts, whereas those lacking these supports enter school already disadvantaged (Koslinski et al., 2022).

Establishing separate norms by school type, however, raises pedagogical and ethical considerations. School-specific norms facilitate the identification of children underperforming relative to peers exposed to comparable environmental stimulation. This process enables the isolation of individual-level difficulty from systemic disadvantage. However, if these criteria are applied without careful consideration, the adoption of lower cutoffs for public school students may potentially lead to the normalisation of widespread academic underachievement. As a result, this could conceal systemic educational failures instead of identifying genuine mathematical learning disabilities (MLDs). This concern aligns with broader evidence of substandard mathematical performance among Brazilian students when evaluated against international standards. Notably, even the upper half of Brazilian students (above the 50th percentile) demonstrated mathematical proficiency that was lower than that of the lower half of students from leading countries in mathematics education, including South Korea, Finland, and Canada, as evidenced by the Programme for International Student Assessment (PISA; OECD, 2023). Separate norms should therefore not be treated as the sole diagnostic criterion, nor replace absolute, curriculum-referenced benchmarks when the goal is to document educational inequality. Beyond this systemic-level safeguard, a key strength of MARKO-S BR lies in its capacity to inform diagnosis and intervention beyond a single summative score. By identifying each child’s specific developmental level and the strategies employed to solve individual items, the instrument enables clinicians and educators to determine targeted intervention points, irrespective of whether the underlying difficulty reflects a genuine mathematical learning disability or insufficient educational exposure.

MARKO-S BR addresses an important gap by demonstrating that screening instruments can be reliably and validly administered digitally to young children, addressing Outhwaite et al.’s (2024) call for research on digital measurement tools in early childhood. The digital administration via tablet-based platform offers practical advantages: pre-recorded audio instructions minimize examiner effects and ensure standardized test presentation across diverse contexts, and the online assessment automates the correction and analysis process. However, important equity considerations warrant discussion. Differential familiarity with digital devices could, in principle, introduce a confounding factor influencing performance on tablet-based assessments, a concern often raised in relation to Brazil’s broader digital divide. However, national survey data indicate that touchscreen device use, primarily via smartphones, is now highly pervasive among Brazilian children across socioeconomic strata, including among lower-income households where smartphones often represent the sole means of internet access (Núcleo de Informação e Coordenação do Ponto BR [NIC.br], 2024), suggesting that basic touchscreen familiarity is less contingent on socioeconomic status than access to computers or broadband internet, which remain more unevenly distributed. Nonetheless, this widespread smartphone familiarity does not necessarily equate to familiarity with the specific tablet device used in the present study, and potential differences in exposure to this assessment format cannot be ruled out. Notably, children do not operate the tablet directly during MARKO-S BR administration, as responses are recorded by the examiner rather than entered by the child, reducing reliance on digital literacy skills such as touchscreen navigation. Implementation in remote or economically disadvantaged regions of Brazil may also face practical barriers related to internet access (Nakayama et al., 2023). Future studies should formally assess children’s prior technology familiarity to more directly rule out its influence in digital assessments.

The present study provided comprehensive validity evidence for MARKO-S BR, a theoretically driven screening instrument, documenting structural validity, reliability, and convergent validity with established mathematical measures, addressing a gap in validity assessments for non-WEIRD populations. The systematic adaptation process ensured the Brazilian version maintains discriminative power across grade levels and school contexts. Future longitudinal research should evaluate the predictive validity of MARKO-S BR scores at school entry in relation to mathematical outcomes in upper elementary grades. Overall, the MARKO-S BR equips educators and psychologists with a psychometrically sound, theoretically grounded screening instrument, supporting early identification of children at risk for MLD, enabling formative assessment to guide targeted interventions.

## Data Availability

The data that support the findings of this study are available on request from the corresponding author.
